# Evaluation of the content quality of websites for recurrent aphthous ulcers and oral lichen planus

**DOI:** 10.1186/s12903-017-0467-1

**Published:** 2017-12-29

**Authors:** Xiaosheng Hu, Hui Pan, Wenxiu He, Hong Hua, Zhimin Yan

**Affiliations:** 1 Department of Oral Medicine, Peking University School and Hospital of Stomatology, 22 Zhongguancun Avenue South, Haidian District, Beijing, 100081 China; 2 Department of Stomotology, Peking University Third Hospital, Beijing, 100091 China

**Keywords:** Recurrent aphthous ulcers, Oral lichen planus, Internet, Website, Oral mucosa

## Abstract

**Background:**

The Internet is one of the most popular resources for people to obtain medical information; however, only a limited number of studies have reported the quality of the available health information related to oral mucosal diseases. The present study aimed to evaluate the quality of information on websites for recurrent aphthous ulcers (RAU) and oral lichen planus (OLP), in both Chinese and English.

**Methods:**

Common search engines, BaiDu, Google, and Yahoo in Chinese; and Bing, Google, and Yahoo in English were used to identify websites providing content related to the oral mucosal diseases. The first 100 links for keywords “recurrent aphthous ulcers” and “oral lichen planus” were visited and content was downloaded within 24 h. Two separate trained researchers use the validated DISCERN rating instrument and JAMA benchmarks to evaluate the content. The rating scores were analyzed and the quality was assessed according to the scores and content of websites.

**Results:**

A total of 145 websites for RAU and 128 of OLP were analyzed. Based on the DISCERN instrument, the quality of the content in websites for both diseases, whether in English or Chinese, was not high, generally scoring 2 to 3 (max. 5). Only 13 of the RAU websites and 21 of the OLP websites fulfilled the four criteria of the JAMA benchmarks. Generally, the scores of the English websites were higher than those of the Chinese websites. During the twelve searches, only four (Yahoo of RAU in Chinese, Bing and Yahoo of RAU in English, and Google of OLP in Chinese) showed moderate correlation between the website’s ranking and their rating scores. People cannot obtain high quality medical information if they only look at the top ranked sites on the viewing lists. Websites belonging to universities or medical centers had relatively higher scores compared with the others.

**Conclusions:**

The quality of the content on websites relating to RAU and OLP in Chinese and English was moderate. More good quality websites and information are needed in the future.

## Background

Oral mucosal diseases include benign conditions, oral infections, immunologically- mediated conditions, potentially malignant disorders, and oral cancers, and involve populations of all ages [1–3]. Recurrent aphthous ulcers (RAU) and oral lichen planus (OLP) are the two of the most common diseases involving the oral mucosa [3]. RAU is a common disorder characterized by recurring ulcers confined to the oral mucosa in patients without other signs of systemic diseases [4]. It affects approximately 20% of the general population, and most patients suffer from varying degrees of pain, which can interfere with speech and eating [3, 4]. Oral lichen planus (OLP) is a chronic inflammatory disease of the oral mucosa with a prevalence of 2% worldwide [5]. The clinical presentations can include reticular, papular, plaque-like, atrophic, erosive, and bullous variants [5]. The World Health Organization (WHO) has defined OLP as a potentially malignant disorder, representing a generalized state associated with a significantly increased risk of oral cancer, with an overall malignant transformation rate reported recently of 1.1% [6]. These two diseases attract a lot of attention from the public and can have large influences on people’s life.

The Internet is one of the most popular resources for people to obtain medical information. People enter key words through a variety of search engines to narrow the scope of information and in return, receive rich content at the tip of their fingers in a matter of seconds. According to the 37th China statistical report on internet development from the China Internet Network Information Center (CNNIC), there were 688 million internet users in China in 2015 and among them, 566 million (82.3%) used search engines, and 22.1% (152 million) were looking for medical information [7, 8]. Patients who consulted the Internet for medical information might change their treatment decisions according to the information on websites [9]. People are worried about the quality of health-related websites, but often forget these concerns once they access a website [10]. Assuming that a high percentage of searches are medical related, and the people performing the searches might be affected by the results, the quality of the medical information contained in websites is critical.

Content published on the Internet is neither regulated nor peer-reviewed, as it is in scientific journals, and is relatively uncontrollable; hence the content may not be accurate or reliable. Tools such as the DISCERN criteria, JAMA score, and health information quality (HIQ) have been used frequently to evaluate the quality of the content on websites [11]. The content information related to “oral leukoplakia” was evaluated and found to be poor and difficult to read [12, 13]. Previous studies investigated the quality of healthcare information related to oral cancer and Sjogren’s syndrome on YouTube, and the information was shown to be less than useful, even misleading [14, 15]. Oral ulcers treatment-related websites identified by the search engine Google were previously assessed according to DISCERN and JAMA benchmarks, and showed variable accuracy levels [16]. Internet information on oral lichen planus accessed using Google and Yahoo Search engines was evaluated by DISCERN and JAMA benchmarks, and the HON code; the overall quality was also shown to be poor [17]. In the present study, the web-based content quality of the two of the most common diseases involving the oral mucosa (RAU and OLP), accessed via three popular searching engines in Chinese (BaiDu, Google, and Yahoo) and English (Bing, Google, and Yahoo) were evaluated. Our analysis was comprehensive because of the higher number of searches performed compared with previous reports, and provided more information to help people choose and use more informative medical information concerning oral mucosal diseases.

## Methods

### Searching and selecting websites

The search engines, three in Chinese, “BaiDu (http://www.baidu.com)”, “Google (http://www.google.com)”, and “Yahoo (http://www.yahoo.com)”; and three in English, “Bing (http://www.bing.com)”, “Google”, and “Yahoo”, were used to identify the websites. The keywords “recurrent aphthous ulcers” and “oral lichen planus” and their corresponding Chinese translated keywords “fu fa xing a fu ta kui yang” and “kou qiang bian ping tai xian” were used in the searches. Two trained evaluators conducted the searching and selection of websites on 19 October 2017, both of whom were dentists. After the respective keywords were used to search using the search engines, the first 100 websites were saved and downloaded as images within 24 h to avoid any changes.

The exclusion criteria for omitting webpages were as follows: 1) Repeated pages, 2) irrelevant information, 3) videos, 4) scholarship articles, and 5) commercial advertisements for drugs or books. All other websites related to the two diseases were included for further analysis.

### Evaluation criteria

The DISCERN and the JAMA benchmarks were chosen as the validated instruments to evaluate the quality of the medical webpages. DISCERN is a brief questionnaire that provides users with a valid and reliable way of assessing the quality of written information on treatment choices for a health problem [18]. The DISCERN handbook is freely downloadable from the DISCERN website [19]. The instrument includes 16 questions, and is organized into three sections: Questions 1–8 addresses the reliability of the publication, which help to consider whether it can be trusted as a source of information about treatment choice; questions 9–15 focus on specific details pertaining to the information relating to treatment alternatives; and question 16 corresponds to the overall quality rating at the end of the instrument [18]. Each question is scored on a scale of 1 to 5 (1 indicates a poor publication and 5 is a good quality publication). Two evaluators (XSH and HP) evaluated the consistency in marking websites using the DISCERN instrument by evaluating the same 30 websites. The weighted Kappa statistic was used to evaluate the agreement of the results between the evaluators. Re-training was applied if the kappa value was less than 0.6. The two evaluators shared the task of reviewing the webpages and made scores for the pages according to the DISCERN instrument. Disagreements were resolved by discussion or by consulting a third reviewer (ZMY or HH).

The JAMA benchmarks were published by the Journal of the American Association, and included four areas: authorship, attribution, disclosure, and currency [20]. One evaluator (WXH) browsed the selected websites and checked whether they display the four areas. The other evaluator (XSH) reviewed the rating. Uncertain ratings were resolved by discussion with a third reviewer (ZMY or HH).

### Statistics

SPSS version 18.0 and SAS was used for data analysis. The agreement between the two evaluators was conducted by the statistics center at Peking University First Hospital. Using SAS, a weighted Kappa statistic of greater than 0.75 was achieved. The t-test and Mann-Whitney Test were to compare the scores of websites between the two languages and the two diseases. Spearman’s correlation test (α = 0.05) was used to analyze the relation between the score and the order of the websites while searching. Chi-Square tests were applied to compare the ratios of the websites meeting each area of the JAMA benchmarks.

## Results

### Analysis of websites from the search results

According to the exclusion criteria, we selected websites from all the searches based on the keywords. For the RAU search in Chinese, the BaiDu search engine yielded 2,970,000 sites, while Google yielded 365,000 and Yahoo yielded 65,000. For the RAU search in English, Bing found 2,090,000 sites, while Google found 70,000 sites, and Yahoo found 3,190,000 sites. For OLP in search in Chinese, BaiDu yielded 2,580,000 sites, Google yielded 728,000, and Yahoo yielded 348,000 websites. For OLP search in English, Bing found 44,700 sites, while Google found 538,000, and Yahoo found 45,500 sites. The first 100 consecutive websites in each search were evaluated. For RAU in Chinese, 77 websites in total were collected (BaiDu 21, Google 35 and Yahoo 21); for ROU in English 68 websites in total were collected (Bing 29, Google 17, and Yahoo 22). For OLP in Chinese, 57 websites in total were collected (BaiDu 15, Google 26, and Yahoo 16); For OLP in English, 71 websites in total were collected (Bing 25, Google 26, and Yahoo 20).

### Assessment of the websites

**Fig. 1:**
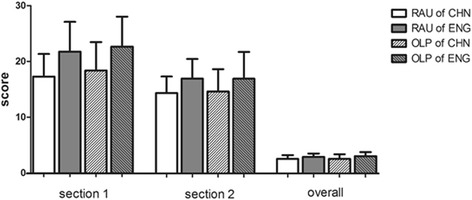
The average scores of websites according to the DISCERN instrument. RAU, recurrent aphthous ulcers; OLP, oral lichen planus; CHN, Chinese; ENG, English

Using the DISCERN questionnaire, the average scores for the 16 questions of each search using either language are shown in Table 1. Most of the sites for both diseases searched in both languages yielded poor quality for Question 1 (Are the aims clear?), 4 (Is it clear what sources of information were used to compile the publication (other than the author or producer)?) and 11 (Does it describe the risks of each treatment?). The overall score (the score of the 16th question) of the sites for both diseases were 2–3, which was a moderate rank. The scores of the two sections and the overall scores of both diseases searching in both languages are shown in Fig. 1.

**Table 1:** Median and mean scores for each question in DISCERN

	RAU of CHN (77) Median /mean	RAU of ENG (68) Median /mean	OLP of CHN (57) Median /mean	OLP of ENG (71) Median /mean
1 aims clear	1/1.03	1/1.24	1/1.00	1
3 relevance	4/4.16	5/4.50	4/4.02	4/4.00
4 source	1/1.38	1/2.24	1/1.72	1/2.23
5 currency	3/2.35	3/2.47	3/2.68	3/3.23
6 balanced and unbiased	4/3.73	4/4.15	4/3.53	4/4.20
7 additional source	2/2.13	5/3.66	2/2.68	5/3.70
8 uncertainty	2/2.44	3/3.12	3/2.74	3/3.39
9 how the treatment works	2/2.13	1/1.50	2/2.11	1/1.59
10 benefits of treatment	2/2.30	3/2.90	2/2.19	2/2.07
11 risks of treatment	1/1.57	1/1.46	1/1.70	1/1.80
12 effects of no treatment	1/1.13	2/2.21	1/1.56	3/2.55
13 effects on life	2/1.95	3/2.38	2/1.91	3/2.28
14 alternative choices	5/4.12	5/4.47	4/3.54	5/4.04
15 shared decision	1/1.09	1/1.97	1/1.56	2/2.54
16 overall quality	3/2.56	3/2.90	3/2.54	3/3.04

*RAU* recurrent aphthous ulcers, *OLP* oral lichen planus, *CHN* Chinese, *ENG* English. The 2nd question of DISCERN was evaluated according to the scores of the 1st question, which cannot be scored when question No.1 scored 1. The table does not cover the outcomes of the 2nd question

For the JAMA benchmarks, six of the RAU searches in Chinese fulfilled the four criteria, and seven in English. For the OLP searches, eight websites in Chinese and 13 in English fulfilled all four criteria. The distribution in each search that fulfilled each criterion of the JAMA benchmarks is presented in Table 2. We found that most websites fulfilled “Disclosure”; however, for “Attribution”, less than half of the websites achieved the criterion.

**Table 2:** Website content based on JAMA benchmarks

JAMA benchmarks	RAU of CHN Number (%)	RAU of ENG Number (%)	OLP of CHN Number (%)	OLP of ENG Number (%)
Authorship	32 (41.6)	37 (54.4)	28 (49.1)	42 (59.2)
Attribution	13 (16.9)	24 (31.2)	13 (22.8)	34 (47.9)
Disclosure	73 (94.8)	66 (97.1)	47 (82.5)	64 (90.1)
Currency	49 (63.6)	34 (50)	31 (54.4)	54 (76.1)

### Differences in content quality of websites in Chinese and English and the two diseases

We compared the scores of the Chinese and English websites for both diseases according to the DISCERN instrument. Generally, English websites scored higher for overall quality score in DISCERN. For both RAU and OLP, the Chinese websites received higher scores for question 9 (which focused on how each treatment works), with a statistically significant difference (*p* < 0.05). Websites in English received significantly higher scores for questions 1, 4, 6, 7, 8, 12, 13, 15, and 16. No differences were found for questions 11 and 14 for both diseases between Chinese and English websites. The scores of each criterion of DISCERN between OLP and RAU websites showed no differences in the same languages, except for questions 14 and 15, in which the OLP showed higher scores for question 15, and RAU showed higher scores for question 14.

We also analyzed the sum of the scores of questions in section 1 (evaluating the reliability of the websites) and section 2 (focusing on specific details of treatment alternatives), and compared the scores in both the diseases and languages and in each search engine. The score for section 1 in the RAU searches in Chinese had an average of 17.27, which was statistically significantly lower than that in English (21.74). In the OLP search, a similar trend was found. The score for the websites in English (16.88) was higher than that for sites in Chinese (14.29). For section 2, English websites also scored more highly than Chinese websites in searches for both diseases. The average score of section 2 for RAU searches in Chinese was 18.37, and in English was 22.65; OLP in Chinese was 14.58, and in English was 16.87. For different search engines, only Yahoo showed a significantly higher score than BaiDu in section 1 assessment and the overall score of OLP searches in Chinese.

When using the four criteria of the JAMA benchmarks, both diseases showed no differences between Chinese and English websites for “Authorship” and “Disclosure”. OLP-related websites in English showed higher ratios for “Attribution” and “Currency” than those in Chinese (*p* < 0.05).

### The categories of websites affecting the scores

**Fig. 2:**
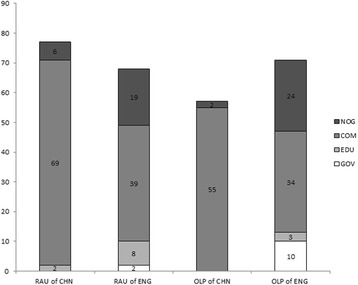
Numbers of websites containing information from different sources. “NOG” was short for non-profit organization, “Gov” for government, “EDU” for university or medical center, “Clin” for clinics or small hospitals, and “COM” for commercial. RAU, recurrent aphthous ulcers; OLP, oral lichen planus; CHN, Chinese; ENG, English

The websites could be divided into four categories according to their source: government, university or medical center, commercial, and non-profit organization. The constitution of websites in each category in each search engine for both diseases is shown in Fig. 2. We found that the websites belonging to universities or medical centers had relatively higher scores than the other kinds of websites. When compared statistically, we found out that websites from universities or medical centers for RAU in Chinese scored significantly higher than commercial websites, both in overall score (3 and 2.54 separately) and score in for section 2 (18 and 16.28 separately). Meanwhile, websites from universities or medical centers for OLP in English were of significantly higher quality than the other types of websites for the section 2 scores of DISCERN.

### The ranking of websites and their overall quality rating scores

We also investigated the correlation between the scores and the ranking of the sites in the view list produced by each search engine. We used the scores for question 16 for the analysis, and the highest overall score was 4. Within websites concerning RAU in English, four websites scored the highest in Google, which were at positions 4, 11, 12, and 71 on the list; the three highest scoring websites in Yahoo were at positions 4, 15, and 21; and in Bing the highest scoring website were at positions 3, 26, and 32. For OLP, 19 websites scored 4, which were at positions 1, 5, 7, 48, 64, and 67 on Google, at 2, 18, 27, 30, 76, and 77 on Yahoo, and at 1, 2, 20, 41, 66, 73, and 76 on Bing. Four Chinese websites in RAU searches (BaiDu position 28, Google position 4 and Yahoo positions 3 and 30) and six of the OLP searches (Bing positions 1 and 12; Google positions 1 and 2; and Yahoo positions 1 and 2) gained 4 points for the 16th question. The Spearman correlation test was used to evaluate the correlation between the sequence in the view list and its corresponding score. We found that the English websites for RAU searched in Bing and Yahoo were moderately correlated, with correlation coefficients of −0.50 and −0.58, respectively; the Chinese websites for RAU in Yahoo and OLP in Google searches were also moderately correlated, with correlation coefficients o − 0.46 and −0.50, respectively.

## Discussion

More and more people would like to get access to the Internet to search for medical information [21]. Incorrect information may lead to unnecessary panic and may play a negative role in people’s decision making [9]. The quality of the websites searched by search engines is essential for people who want information on the diseases.

A limited number of studies have been published about the quality of the health information available on the Internet related to oral mucosal diseases. In this study we focused on the quality of websites related to RAU and OLP and the difference in the information available in Chinese and in English.

In our research, we used the DISCERN questionnaire and JAMA benchmarks. Overall, the quality of the websites identified for RAU and OLP was moderate, which agreed with previous studies of oral lichen planus and oral ulcers [16, 17]. Comparing the websites in English with those in Chinese, we found that the websites in English obtained higher scores. We found that there were some differences between the English and Chinese narration. For example, for question 9: “Does it describe how each treatment works?”, websites in Chinese got relatively higher scores; while for question 4 about the sources of the information used in the publication, websites in English had an advantage. In the authors’ opinion, the differences in expressions between the two languages might have played a part in these differences. Chinese websites stress advantages, but do not pay the same amount of attention to the resources they provide. People searching for medical information in their native language might be biased toward their wants and thoughts. In addition, for some websites, the information itself was correct from a doctors’ perspective; however, they got low scores because of the partial introduction of the diseases.

RAU and OLP related information in the websites in the same language showed similar scores. According to the DISCERN instrument, both Chinese and English websites for OLP showed higher scores for question 15 (Does it provide support for shared decision-making?), and RAU-related websites showed higher scores for question 14 (Is it clear that there may be more than one possible treatment choice?). From our perspective, this was because of the different characteristics of the two diseases. In other words, OLP is a potentially malignant disease, and people pay more attention to cancer transformation information and shared decision making; whereas RAU affects a larger population, and people tend to seek more treatment choices.

It is impossible to scan all the sites that are identified by a search engine, so the websites ranked in the top part of the view list are more likely to be seen by searchers, thus, their content quality is crucial to the public. People usually only read the first page of a search engine list; it was reported that more than 70% only viewed the first five links [22]. Our research showed that during the 12 searches, only four (Yahoo for RAU in Chinese, Bing and Yahoo for RAU in English, and Google for OLP in Chinese) showed moderate correlation between the ranking of the websites and their rating scores. High quality websites did not always rank in the top part of the list in the search engines when using different search keywords. This means that people cannot obtain relatively high quality medical information if they only look at the first page of a search engine search list.

Different sources of websites showed different qualities. Websites from universities or medical centers usually had higher quality scores, and supply very important information for people. Thus, accessing these types of sites could provide people with better information of oral health. In the future, people controlling the medical education content in websites should focus on the accuracy, reliability, and readability of the content, and are recommended to refer to validated tools, such as the DISCERN criteria, JAMA score, and health information quality (HIQ).

Our research focused on the websites in the popular search engines for the two common diseases involving the oral mucosa. BaiDu is the most commonly used search engine in Mainland China, which partly reflects the medical information most people could obtain in Mainland China [8]. Google and Yahoo are popular worldwide. Thus, we believe that our study is representative of the web-searching activities of people in Mainland China and also reflects international conditions.

The Internet is dynamic, and the search results change all the time. We recorded the websites on 1 day, and they may not be representative of the information available at a later date. We used the DISCERN equipment to measure and compare the quality of the information, which focuses on treatment, and thus may have certain limitations and cannot reflect other facets of the disease.

## Conclusions

According to the DISCERN questionnaire and JAMA benchmarks, the quality of the websites relating to ROU and OLP in Chinese and English is moderate. In addition, there are some differences between Chinese and English health information in the websites. Websites from universities or medical centers are usually of higher quality. More good quality websites and information are in needed in future.

## Abbreviations

OLP: Oral lichen planus
RAU: Recurrent aphthous ulcers
